# Structural Characterization and Anti-Gouty Nephropathy Potential of Polysaccharides from *Atractylodes chinensis*

**DOI:** 10.3390/molecules30040757

**Published:** 2025-02-07

**Authors:** Xue Chen, Ruipu Jia, Kai Zhang, Shiqing Sun, Mei Mei, Hong Zhao, Yu Shen, Yuliang Wang, Yu Zhang

**Affiliations:** 1 College of Pharmacy, Jiamusi University, Jiamusi 154007, China; 19997560715@163.com (X.C.); jiaruipu2000@163.com (R.J.); 0316zh@163.com (H.Z.); shenyu@jmsu.edu.cn (Y.S.); 2 School of Basic Medicine, Jiamusi University, Jiamusi 154007, China; zhangkai930222@163.com (K.Z.); ssq19971030@163.com (S.S.); 15645091129@163.com (M.M.); 3 Heilongjiang Provincial Key Laboratory of New Drug Development and Pharmacotoxicological Evaluation, Jiamusi University, Jiamusi 154007, China

**Keywords:** *Atractylodes chinensis* (DC.) Koidz., gouty nephropathy, polysaccharide, NLRP3, structural characterization

## Abstract

Polysaccharides derived from *Atractylodes chinensis* (DC.) Koidz. (ACP), a traditional Chinese medicine, were extracted and analyzed for their structural characteristics and anti-gouty nephropathy (GN) activity. Sprague-Dawley (SD) rats were divided into six groups: control, model, positive control, and three treatment groups (ACP-60-L, ACP-60-M, and ACP-60-H). Treatment significantly reduced inflammatory responses and renal damage, as evidenced by decreased levels of uric acid (UA), creatinine (Cr), and blood urea nitrogen (BUN), alongside modulation of NOD-like receptor protein 3 (NLRP3) expression in renal tissues. ACP-60 was fractionated into three polysaccharides, including ACP-60-A (Mw 9.18 kDa), ACP-60-B (Mw 58.21 kDa), and ACP-60-C (Mw 109.01 kDa) using DEAE-52 cellulose column chromatography. Monosaccharide analysis revealed that ACP-60-A predominantly comprised fructose (Fru) and glucose (Glc), ACP-60-B contained rhamnose (Rha), galactose (Gal), Fru, and mannose (Man), and ACP-60-C included Man, Gal, Rha and xylose (Xyl). In vitro studies using HK-2 cells confirmed the anti-GN activity of all three fractions, with ACP-60-A demonstrating the highest efficacy. Structural elucidation of ACP-60-A identified its main glycosidic linkages as a →1)-β-Fru*f*-(2→ backbone with α-Glc*p*-(1→ and β-Fru*f*-(2→ branches. The underlying mechanism of ACP-60-A’s anti-GN activity is associated with inhibition of the NLRP3 inflammasome signaling pathway, suppression of downstream inflammatory factor release, and downregulation of NLRP3, ASC, and Caspase-1 protein expression. Further studies demonstrated that the superior activity of ACP-60-A is attributable to its lower molecular weight, specific monosaccharide composition, and unique glycosidic bond arrangement. ACP-60-A shows potential for increased anti-GN efficacy through purification or modification, laying the groundwork for developing novel therapeutic agents for GN.

## 1. Introduction

Gouty nephropathy (GN) is primarily caused by elevated uric acid (UA) levels due to dysregulated purine metabolism, resulting in the deposition of sodium urate crystals in renal tubules [1]. The pathogenesis of GN involves complex mechanisms, including inflammatory responses, endothelial dysfunction, and fibrosis [2]. The NLRP3 inflammasome, along with its adaptor protein ASC (apoptosis-associated speck-like protein containing a CARD) and the effector molecule Caspase-1, has been extensively studied in recent years for its role in inflammation and autoimmune diseases. Sodium urate crystals deposited in vivo serve as danger signals that activate the NLRP3 inflammasome, leading to the conversion of pro-Caspase-1 into its active form, Caspase-1. Caspase-1, in turn, facilitates the maturation and secretion of pro-inflammatory cytokines interleukin-1β (IL-1β) and interleukin-18 (IL-18). Notably, IL-1β has been implicated as a key driver of systemic inflammation, exacerbating renal injury in GN [3,4].

The treatment options for gouty nephropathy at this stage are mainly symptomatic, such as inhibition of UA synthesis and promoting its excretion, lowering uric acid levels, improving blood circulation, and reducing inflammation levels. This further reduces the risk of developing metabolic syndromes, such as hypertension, diabetes, and cardiovascular disease, thereby improving the overall health status of the patient. However, these drugs often carry significant adverse effects, including allergic reactions and hepatotoxicity [5]. Traditional Chinese medicine has gained prominence as an alternative therapy for chronic diseases due to its efficacy and reduced side effect profile. Traditional Chinese medicine offers unique advantages in managing GN by slowing disease progression, mitigating inflammation, and aiding in the restoration of organ function [6]. Youguiyin, a traditional herbal compound used for the treatment of nephropathy, could effectively inhibit NF-κB expression and ROS levels as well as improve Scr and UA levels in high yeast-induced GN rats, thus showing a nephroprotective effect [7]. Some studies have reported that utilizing extracts of some plants can also significantly lower uric acid levels, reduce the deposition of urate crystals in the kidneys of mice, and improve kidney function [8]. Since there is a lack of effective drugs to treat GN, it is imperative to develop efficient new drugs and better treatment strategies.

*Atractylodes chinensis* (DC.) Koidz., a perennial herb in the family Asteraceae, is widely recognized for its medicinal applications. Its dried rhizome is a traditional herbal remedy with documented anti-tumor, anti-inflammatory, antioxidant, and immunomodulatory properties. It has been utilized in the treatment of digestive and inflammatory disorders and is approved for use as a functional food [9,10]. The herb, known as *Cangzhu*, is listed in the Chinese Pharmacopoeia (2020 edition) as the dried root of either *Atractylodes lancea* (Thunb.) DC. or *Atractylodes chinensis* (DC.) Koidz., and is commonly employed in the management of gastrointestinal disorders. Additionally, it exhibits diuretic and analgesic effects [11,12]. Historically, *Cangzhu* has also been included in compound formulations for treating GN, although the precise bioactive constituents and mechanisms of action remain unclear and warrant further investigation. Currently, the principal bioactive components extracted from *Atractylodes chinensis* include polysaccharides, volatile oils, and glycosides, with polysaccharides being the predominant class. While research on *Atractylodes chinensis* has primarily focused on its small molecule compounds, studies investigating its polysaccharides are scarce. Polysaccharides are high-molecular-weight carbohydrates linked by glycosidic bonds and are abundantly present in nature [13,14]. In traditional Chinese medicine, polysaccharides frequently constitute the primary active ingredients due to their immunomodulatory, anti-inflammatory, hepatoprotective, and antioxidant activities. Their ease of extraction, low toxicity, and minimal side effects have garnered significant research interest [15,16,17,18].

In this study, sub-fractions were extracted using graded ethanol, and the optimal fraction was identified through screening with HK-2 cells to investigate anti-GN activity and underlying mechanisms. The optimal fraction was further purified using DEAE-52 cellulose column chromatography, and its structural characteristics were analyzed. Subsequently, the structure-activity relationship and anti-GN effects were validated in HK-2 cells. The findings of this study may contribute to the development of novel, effective, and low-toxicity therapeutic agents for GN.

## 2. Results

### 2.1. Extraction and Isolation of Polysaccharides

Crude polysaccharides were extracted using hot water extraction, centrifugation, ethanol precipitation, and lyophilization. Subsequently, graded ethanol precipitation was employed to obtain different fractions. The extraction rate and sugar content of the six fractions are summarized in Table 1. Among the fractions, ACP-70 exhibited the highest extraction rate, followed by ACP-60, while ACP-30 had the lowest extraction rate. Notably, ACP-60 demonstrated the highest sugar content at 77.21 ± 2.41%.

ACP-60 was identified as the optimal component based on its superior sugar content and extraction rate. In preliminary experiments using HK-2 cells, the cell survival rate was assessed via the CCK-8 assay, cell morphology was observed, and NLRP3, ASC, and Caspase-1 protein expression were analyzed to evaluate the anti-GN effects of the polysaccharide fractions. The results indicated that ACP-60 exhibited the highest activity, leading to the selection of ACP-60 for subsequent studies.

### 2.2. Purification, Molecular Weight and Monosaccharide Composition of ACP-60

**Figure 1:**
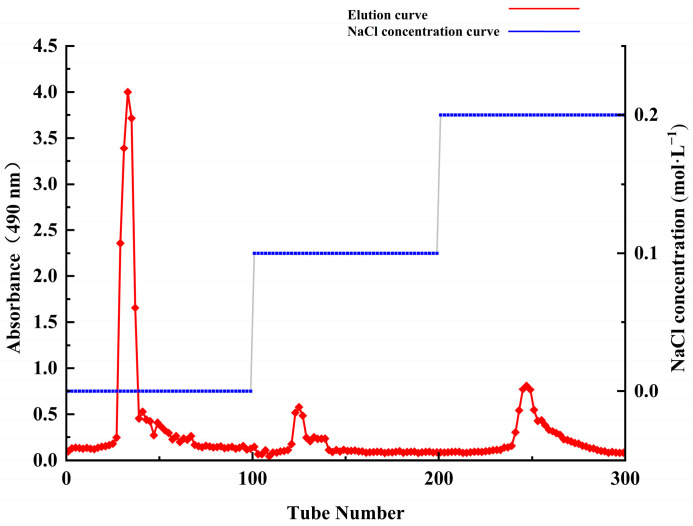
Elution curves of ACP-60-A, ACP-60-B, and ACP-60-C on DEAE-52 cellulose column.

**Figure 2:**
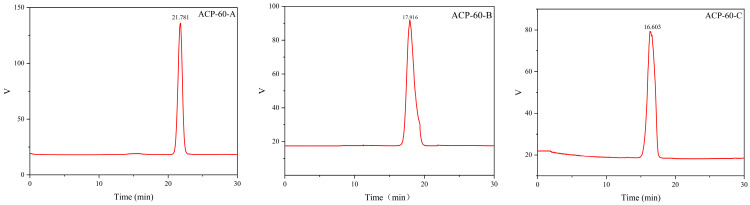
Molecular weights of ACP-60-A, ACP-60-B, and ACP-60-C.

**Figure 3:**
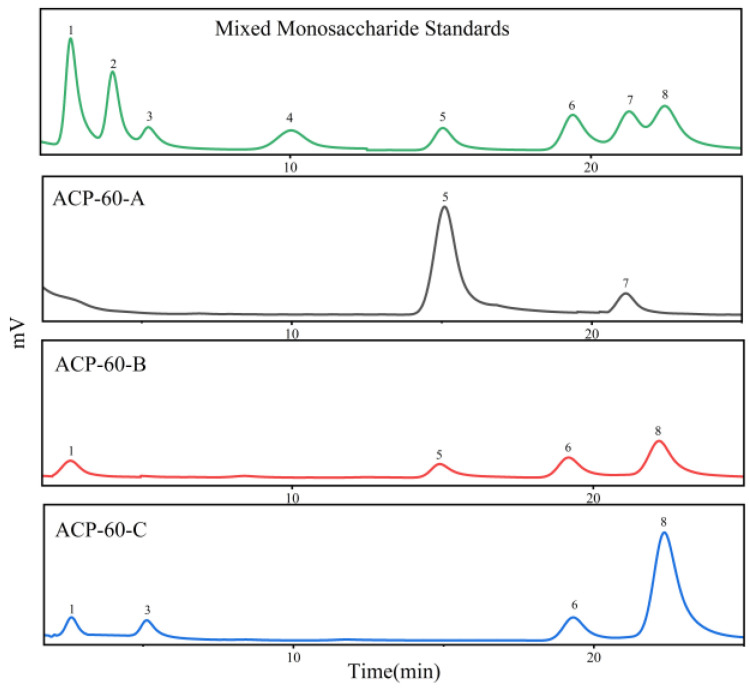
Analysis of monosaccharide composition by HPLC for ACP-60-A, ACP-60-B, and ACP-60-C. A: mixed monosaccharide standards 1. Rha; 2. Fuc; 3. Xyl; 4. Ara; 5. Fru; 6. Man; 7. Glc; 8. Gal; B: ACP-60-A; C: ACP-60-B; D: ACP-60-C.

As shown in Figure 1, ACP-60 was purified using the DEAE-52 column, yielding three sub-fractions (ACP-60-A, ACP-60-B, and ACP-60-C). The three homogeneous polysaccharides obtained were ACP-60-A (50.76 ± 1.21%, wt%), ACP-60-B (2.58 ± 0.83%, wt%), and ACP-60-C (5.40 ± 1.09%, wt%). These polysaccharides exhibited a single, symmetrical peak, as shown in Figure 2. The elution times for ACP-60-A, ACP-60-B, and ACP-60-C were approximately 21.781, 17.916, and 16.603 min, respectively. Based on the standard dextran calibration curve (log Mw = −0.2075x + 8.4826, R^2^ = 0.9903), the average molecular weights of ACP-60-A, ACP-60-B, and ACP-60-C were calculated to be 9.18 kDa, 58.21 kDa, and 109.01 kDa, respectively. The monosaccharide composition analysis of ACP-60-A, ACP-60-B, and ACP-60-C is shown in Figure 3. Comparison of the retention times with mixed monosaccharide standards revealed that ACP-60-A is primarily composed of Glc and Fru, ACP-60-B contains Rha, Gal, Fru, and Man, and ACP-60-C is composed of Man, Rha, Gal, and Xyl. The monosaccharide composition and molarity of ACP-60-A, ACP-60-B, and ACP-60-C are detailed in Table 2.

Previous studies have reported that a neutral polysaccharide (AP) with Mw of 60.61 kDa derived from *Atractylodes chinensis* (DC.) Koidz. was primarily composed of Gal and Ara [19]. Moreover, studies have also shown that the AKU monosaccharides extracted from *Atractylodes chinensis* (DC.) Koidz. were mainly composed of Ara, Gal, Glc, Rha, GalA, and GlcA [20]. Additionally, AKP1 had an average Mw of 3.25 kDa and was composed of Rha, Ara, and Gal in a molar ratio of 1:1.25:2.88 [21]. The polysaccharides extracted in the present study were different from those reported previously. Fru was found in ACP-60-A and ACP-60-B, and there was higher content in ACP-60-A. Therefore, these results suggested that the different activities of polysaccharides from *Atractylodes chinensis* (DC.) Koidz. might be related to the molecular weight and monosaccharide composition.

### 2.3. FT-IR Spectra

**Figure 4:**
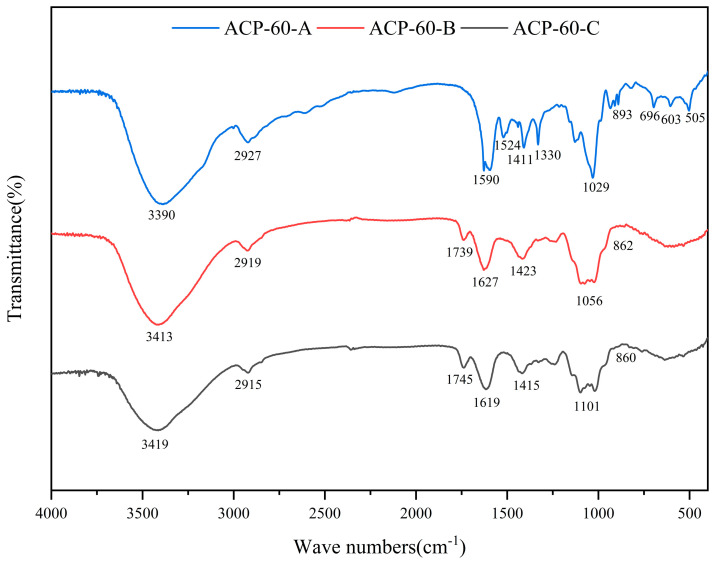
FT-IR spectra of the polysaccharide fractions ACP-60-A, ACP-60-B, and ACP-60-C.

Figure 4 represents the FT-IR spectra of ACP-60-A, ACP-60-B, and ACP-60-C. The broad band centered in 3390–3419 cm^−1^ was attributed to the O–H stretching vibration. The next narrower band at 2915–2927 cm^−1^ was attributed to C–H stretching vibration. Two bands at 1590–1627 cm^−1^ and 1411–1423 cm^−1^ arose from antisymmetric and symmetric stretching vibrations of uronic carboxylate groups COO–, respectively. Furthermore, the absorption band 1029–1101 cm^−1^ was assigned to the stretching vibration of the C-O bond [22,23,24]. These bands indicated the presence of polysaccharides and exhibited a high degree of similarity. For ACP-60-B and ACP-60-C, the band at 1739–1745 cm^−1^ represented the C=O stretching vibration of the ester or free carboxylic groups COOR/COOH. The absorption band in the spectrum of ACP-60-A at 1524 cm^−1^ was characteristic of the amide II vibration associated with proteins and was produced by N-H bending vibration. There are several narrow and intense bands at 505–696 cm^−1^, which may be polyphenols [25,26]. The absorption bands at 890 cm^−1^ and 860 cm^−1^ suggested the presence of β- and α-glycosidic bonds in ACP-60-A, ACP-60-B and ACP-60-C, respectively.

### 2.4. Methylation Analysis

The methylation analysis results of ACP-60-A are listed in Table 3. The types of glycosidic bonds in ACP-60-A were further investigated by analyzing the methylation products. The glycosidic residues corresponding to each peak were identified based on the relative retention time and mass spectrometry results. Three glycosidic bonds were identified in ACP-60-A: Glc*p*-(1→, Fru*f*-(2→ and →1)-Fru*f*-(2→, with the relative molar ratios of 15.90:23.38:60.72.

Overall, the findings from the monosaccharide analysis and methylation results were consistent. The anti-GN properties may be associated with the specific configuration of the glycosidic linkages in ACP-60-A.

### 2.5. NMR Analysis of ACP-60-A

**Figure 5:**
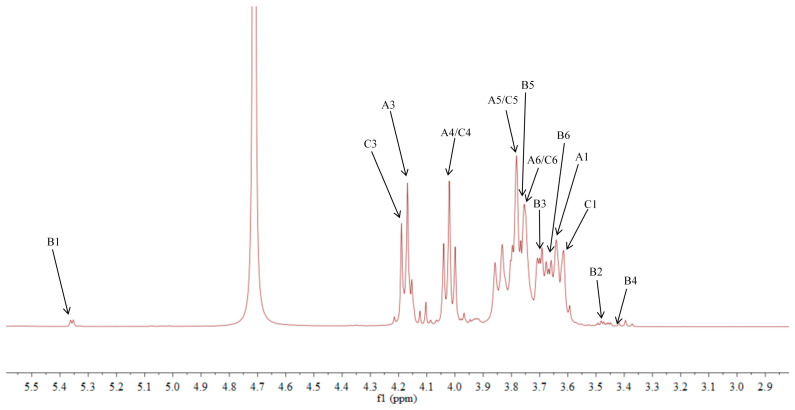
^1^H NMR spectra of ACP-60-A.

**Figure 6:**
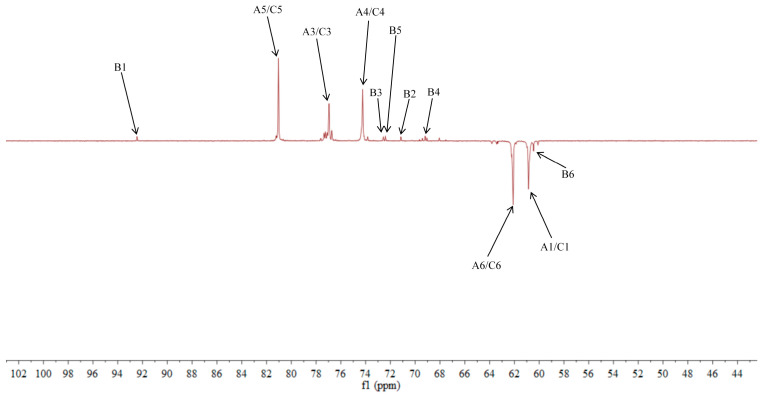
DEPT-135 spectra of ACP-60-A.

**Figure 7:**
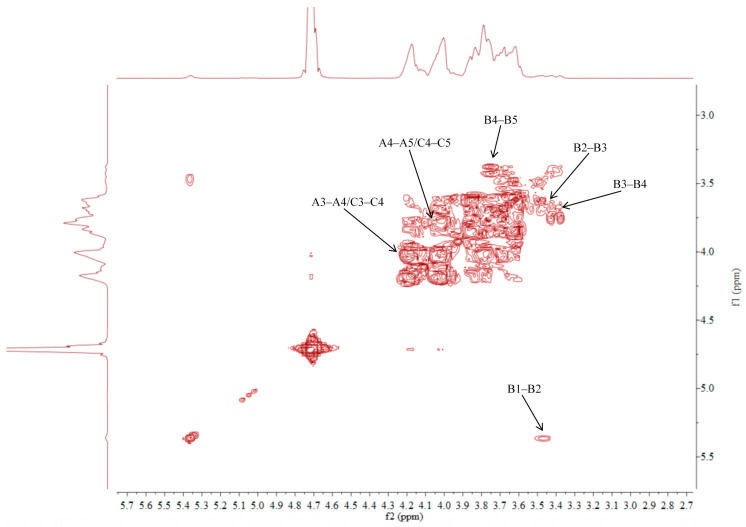
^1^H-^1^H COSY spectra of ACP-60-A.

**Figure 8:**
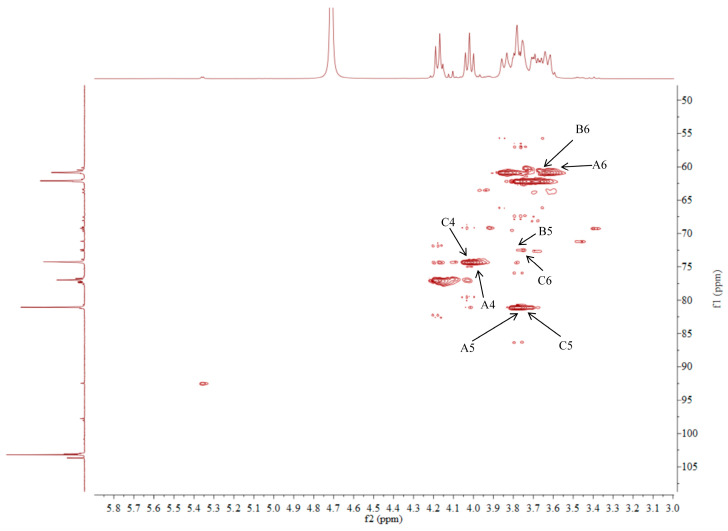
HSQC spectra of ACP-60-A.

**Figure 9:**
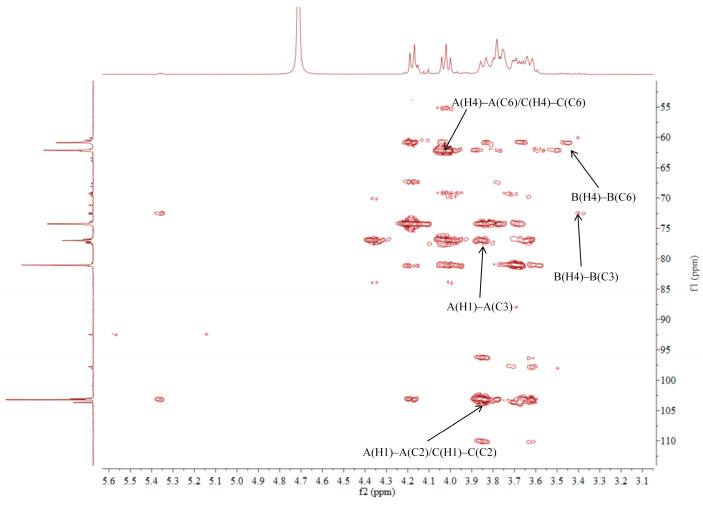
HMBC spectra of ACP-60-A.

**Figure 10:**
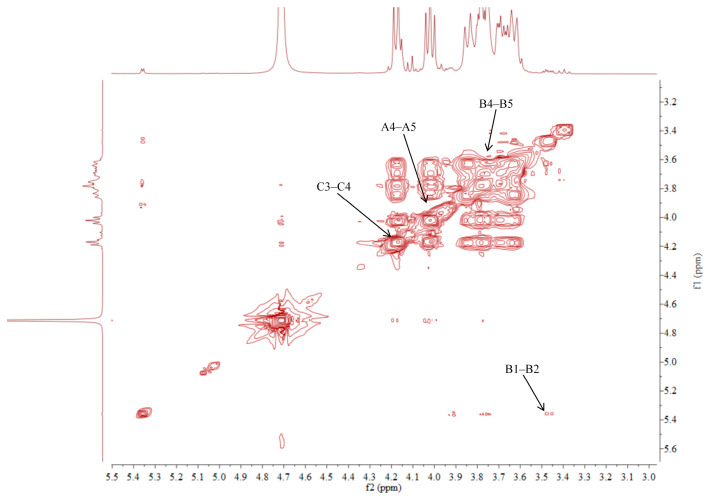
NOESY spectra of ACP-60-A.

The ^1^H NMR spectrum of ACP-60-A displayed an anomeric proton signal at low-field δ_H_ 5.36. Based on the analysis of the monosaccharide composition and methylation results, δ_H_ 5.36 was presumed to correspond to the end-group hydrogen signal of α-Glc*p*-(1→, with the remaining signals appearing primarily in the range δ_H_ 3.61–4.19. The ^13^C NMR spectra revealed that δ_C_ 103.2 was a heterocapitate carbon signal, and δ_C_ 60.4–81.1 corresponded to glycocyclic carbon signals linked to oxygen [27]. Hydrocarbon signals from the sugar residues were attributed to each sugar component by analyzing the ^1^H NMR, ^13^C NMR, DEPT-135, ^1^H-^1^H COSY, HSQC, HMBC, and NOESY spectra, as summarized in Figure 5, Figure 6, Figure 7, Figure 8, Figure 9 and Figure 10 and Table 4.

The δ_C_ 92.46 and δ_H_ 5.36 ppm signals are characteristic of the end-group carbon-hydrogen signal of α-Glc*p*-(1→, and further analysis of H2-H5 using ^1^H-^1^H COSY provided chemical shift values of 3.45, 3.68, 3.39, and 3.76, respectively [28]. The HMBC spectra revealed that H4 correlated with C 72.58, 71.16, and 60.45, with δ_C_ 60.45 being identified as the C6 signal of α-Glc*p*-(1→. δ_C_ 103.21 represents the typical end-group C2 signal of →1)-β-Fru*f*-(2→, while δ_C_ 103.67 corresponds to the end-group C2 signal of β-Fru*f*-(2→ In the DEPT-135 spectrum, δ_C_ 60.87 is attributed to –CH_2_, which forms a -CH_2_O-group with δ_H_ 3.85 and 3.62, and the HMBC spectrum showed that δ_H_ 3.85 correlated with δ_C_ 103.21, 103.67, and 76.97, establishing that δ_C_ 60.87 is a C1 signal and δ_C_ 76.97 is a C3 signal. The ^1^H-^1^H COSY spectrum showed that H3 (δ_H_ 4.18) correlated with δ_H_ 4.02, indicating that δ_H_ 4.02 is the H4 signal. H4, in turn, correlated with δ_H_ 3.77, identifying δ_H_ 3.77 as the H5 signal. The HMBC spectrum analysis of H4 and C 62.11 confirmed that C 62.11 corresponds to the C6 signal [29].

The NMR results indicate that ACP-60-A is primarily composed of α-Glc*p*-(1→, β-Fru*f*-(2→, and →1)-β-Fru*f*-(2→, with the →1)-β-Fru*f*-(2→ being more abundant. It is hypothesized that the main chain of ACP-60-A consists of →1)-β-Fru*f*-(2→, with α-Glc*p*-(1→ and β-Fru*f*-(2→ linking the structure. Based on the results of monosaccharide composition, methylation, and NMR spectroscopy, it was found that these polysaccharides were different from those reported previously. Previous studies have reported that AP consisted of mainly →3,6)- α-D-Gal*p*-(1→, with the side chains of →5)-α-L-Ara*f*-(1→ and α-L-Ara*f* [18]. Therefore, the present study mainly elucidated the structural features of ACP-60-A.

### 2.6. Effects of ACP-60-A, ACP-60-B and ACP-60-C on HK-2 Cell Viability

**Figure 11:**
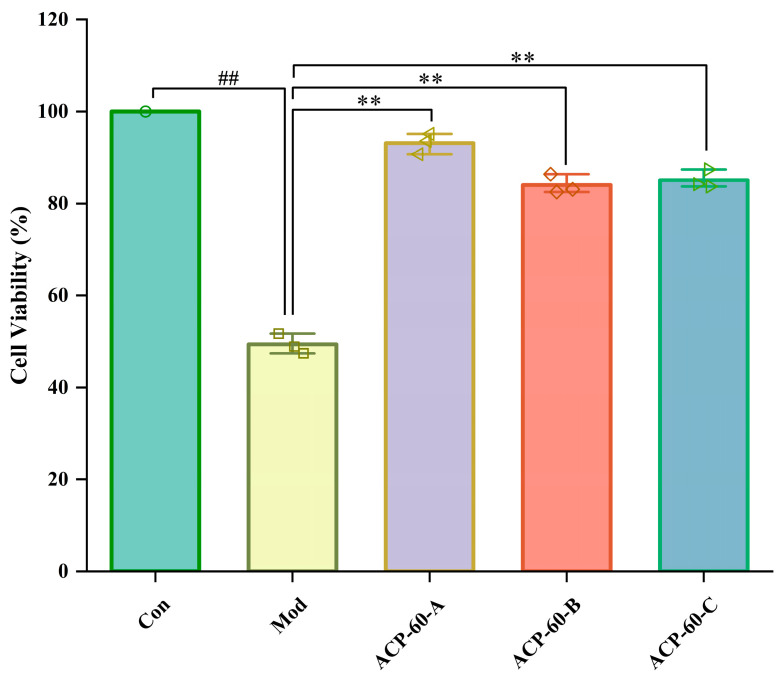
Effects of ACP-60-A, ACP-60-B, and ACP-60-C on the survival rate of HK-2 cells. ($\overset{-}{x}$ ± s; *n* = 3) ^##^ *p* < 0.01 vs. the Con group, ** *p* < 0.01 vs. the Mod group.

As shown in Figure 11, the survival rate of HK-2 cells in the Mod group significantly decreased compared to the Con group, indicating a highly significant difference (*p* < 0.01). In contrast, the survival rates of HK-2 cells in the ACP-60-A, ACP-60-B, and ACP-60-C groups all increased, with the difference being highly significant (*p* < 0.01). These results demonstrate that ACP-60-A, ACP-60-B, and ACP-60-C can significantly enhance HK-2 cell viability, with ACP-60-A showing the highest efficacy in improving cell survival.

### 2.7. Observation of HK-2 Cells Morphology

**Figure 12:**
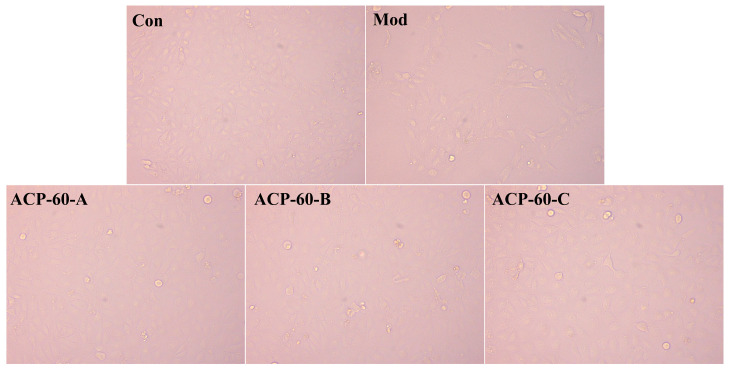
Plot of the morphology of HK-2 cells (100×).

The Con group exhibited healthy cells, which were well-aligned and orderly, as shown in Figure 12. After 36 h of UA stimulation, HK-2 cells became elongated, deformed, and disorganized, with noticeable fibrosis. Following treatment with ACP-60-A, ACP-60-B, and ACP-60-C, the cell morphology of each group improved, with an increased number of adherent cells. Some cells regained a more normal shape and were more orderly arranged, and fibrosis was significantly reduced. These results suggest that ACP-60-A, ACP-60-B, and ACP-60-C significantly improved the HK-2 cell morphology, with ACP-60-A showing the highest efficacy.

### 2.8. Protein Expression of NLRP3, ASC, and Caspaes-1 in HK-2 Cells

**Figure 13:**
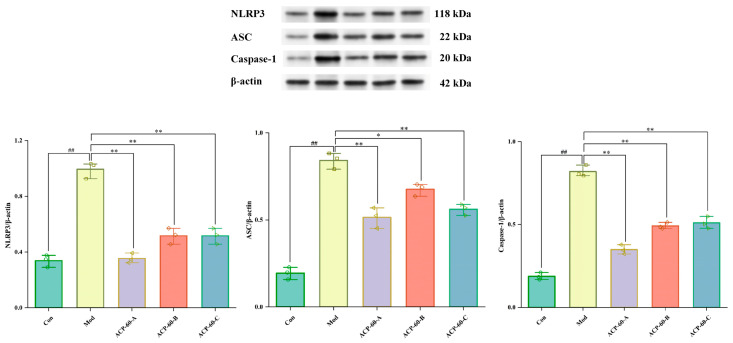
Expression of NLRP3, ASC, and Caspase-1 proteins in HK-2 cells. ($\overset{-}{x}$ ± s; *n* = 3) ^##^ *p* < 0.01 vs. the Con group, * *p* < 0.05, ** *p* < 0.01 vs. the Mod group.

As shown in Figure 13, the protein expression of NLRP3, ASC, and Caspase-1 in the Mod group was significantly higher (*p* < 0.01) compared to the Con group. In contrast, the protein expression of these markers in the ACP-60-A and ACP-60-C groups was significantly decreased (*p* < 0.01) relative to the Mod group. In the ACP-60-B group, the expression of NLRP3 and Caspase-1 was significantly reduced (*p* < 0.01), while ASC expression was also significantly reduced (*p* < 0.05) when compared to the Mod group. Overall, ACP-60-A, ACP-60-B, and ACP-60-C all significantly decreased the protein expression of NLRP3, ASC, and Caspase-1. Among these, ACP-60-A exhibited the most pronounced inhibitory effect.

### 2.9. Effects of ACP-60 on 24-h Urinary Protein Levels in GN Rats

**Figure 14:**
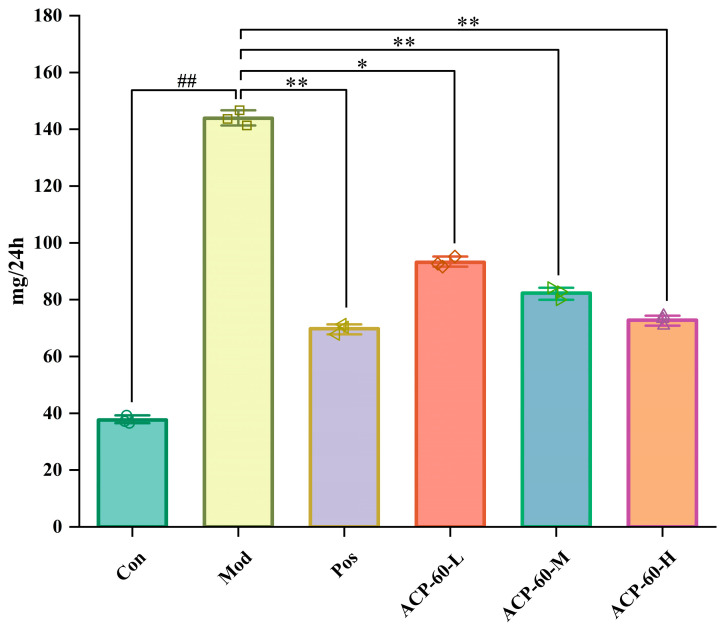
Effect of ACP-60-L, M, H on the urinary protein content of GN rats. ($\overset{-}{x}$ ± s; *n* = 3), ^##^ *p* < 0.01 vs. the Con group, * *p* < 0.05, ** *p* < 0.01 vs. the Mod group.

Urinary protein serves as an important biomarker for kidney disease diagnosis and treatment. In the current study, the 24-h urinary protein levels in the Mod group were significantly elevated compared to the Con group (*p* < 0.01), confirming the successful establishment of the GN model. In comparison with the Mod group, the Pos group and the medium- and high-dose ACP-60 groups (ACP-60-M and ACP-60-H) showed a highly significant reduction in urinary protein levels (*p* < 0.01), while the low-dose ACP-60 group (ACP-60-L) demonstrated a significant decrease (*p* < 0.05) (Figure 14). These findings suggest a dose-dependent effect of ACP-60 on reducing urinary protein excretion. Notably, the high-dose ACP-60 (ACP-60-H) was particularly effective, indicating its potential to protect kidney function and delay the progression of kidney disease.

### 2.10. Effects of ACP-60 on Serum Biochemical Parameters Levels in GN Rats

**Figure 15:**
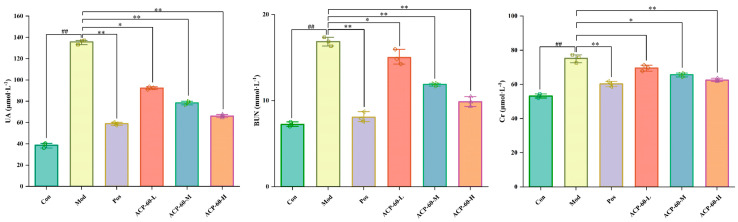
Effect of ACP-60-L, M, H on UA, Cr, BUN content of GN rats. ($\overset{-}{x}$ ± s; *n* = 3) ^##^ *p* < 0.01 vs. the Con group, * *p* < 0.05, ** *p* < 0.01 vs. the Mod group.

The serum levels of UA, Cr, and BUN were significantly elevated in the Mod group compared to the Con group (*p* < 0.01), as shown in Figure 15. Treatment with allopurinol (Pos group) and medium- and high-dose ACP-60 (ACP-60-M and ACP-60-H groups) resulted in significant or highly significant reductions in these parameters (*p* < 0.05 or *p* < 0.01). The low-dose ACP-60 group (ACP-60-L) exhibited a significant trend of reduction in UA, Cr, and BUN levels, although some changes were not statistically significant (*p* < 0.05). The observed effects were dose-dependent, with the high-dose ACP-60 (ACP-60-H) showing the most pronounced improvement in renal function. UA, Cr, and BUN are critical biomarkers for assessing kidney function, and the reductions observed following ACP-60 treatment indicate its potential efficacy in improving renal health and mitigating kidney damage.

### 2.11. Effects of ACP-60 on IL-18 and IL-1β Levels in GN Rats

**Figure 16:**
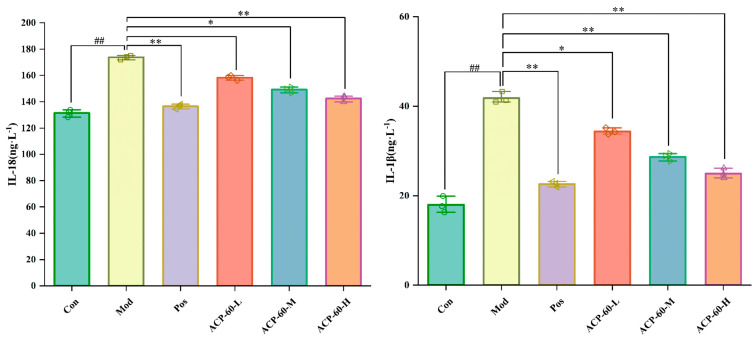
Effect of ACP-60-L, M, H on IL-18, IL-1β content of GN rats. ($\overset{-}{x}$ ± s; *n* = 3) ^##^ *p* < 0.01 vs. the Con group, * *p* < 0.05, ** *p* < 0.01 vs. the Mod group.

The levels of IL-18 and IL-1β in kidney tissue were significantly elevated in the Mod group compared to the Con group (*p* < 0.01), as shown in Figure 16. Treatment with allopurinol (Pos group) and high-dose ACP-60 (ACP-60-H) significantly reduced these inflammatory markers (*p* < 0.01). Moderate-dose ACP-60 (ACP-60-M) also resulted in significant or highly significant decreases (*p* < 0.05 or *p* < 0.01), while low-dose ACP-60 (ACP-60-L) showed a significant trend of reduction or no significant change (*p* < 0.05). The reductions in IL-18 and IL-1β levels demonstrated a dose-dependent pattern. As key mediators of systemic inflammation, IL-18 and IL-1β are implicated in exacerbating kidney injury. The results indicate that ACP-60, particularly at high doses, effectively mitigates kidney inflammation and injury, with ACP-60-H exhibiting the most potent anti-GN activity.

### 2.12. Histological Analysis (HE Staining)

**Figure 17:**
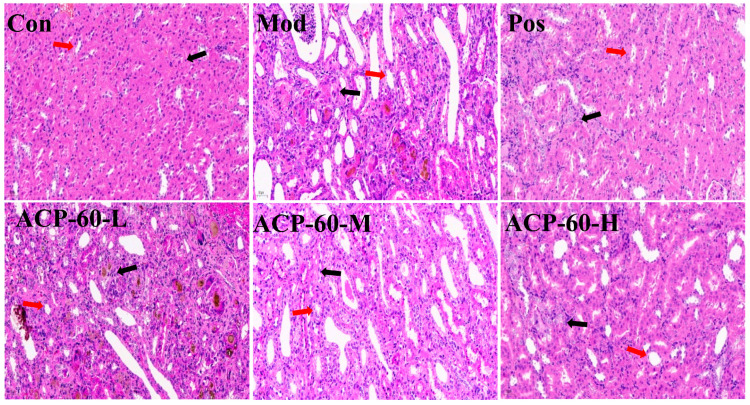
Pathological observation of renal tissue (HE staining, 400×). The glomerulus and tubules are individually marked by black and red arrows.

Histological observations of renal tissues revealed that the Con group displayed well-organized renal tubules and intact glomeruli, with no signs of necrosis, fibrosis, or inflammatory infiltration (Figure 17). In contrast, the Mod group showed significant renal damage, including dilated renal tubules, disrupted glomerular structures, and extensive inflammatory infiltration. Treatment with allopurinol (Pos group) and high-dose ACP-60 (ACP-60-H) significantly mitigated these pathological changes, as evidenced by reduced glomerular necrosis, decreased tubular edema, and diminished inflammatory infiltration. Moderate-dose ACP-60 (ACP-60-M) and low-dose ACP-60 (ACP-60-L) also improved glomerular necrosis and inflammatory markers, though the effects were less pronounced compared to the high-dose group. The results underscore the potent therapeutic effects of ACP-60, particularly at high doses, in alleviating glomerular necrosis and inflammation, ultimately improving renal function. ACP-60-H exhibited the most substantial anti-GN efficacy among the treatment groups.

### 2.13. Protein Expression of NLRP3, ASC, and Caspaes-1 in Renal Tissue

The deposition of sodium urate crystals is a key contributor to GN. Sodium urate acts as a danger signal in vivo, activating the NLRP3/ASC/Caspase-1 signaling pathway, which plays a pivotal role in inflammation and kidney damage. Previous studies, such as those on Plantaginis Semen polysaccharides (PSP), have demonstrated the use of this pathway in treating nephropathy and mitigating renal injury [5].

**Figure 18:**
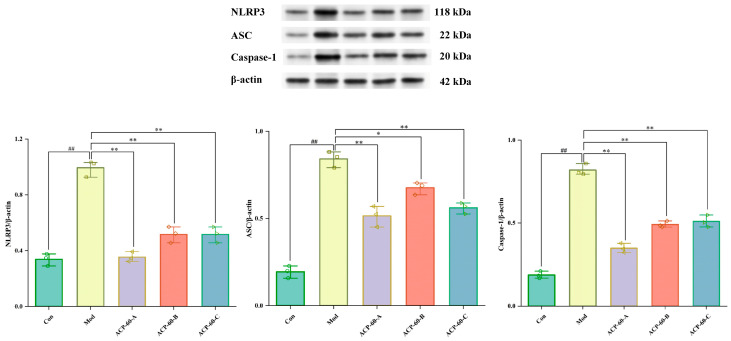
Expression of NLRP3, ASC, and Caspaes-1 proteins in renal tissue. ($\overset{-}{x}$ ± s; *n* = 3) ^##^ *p* < 0.01 vs. the Con group, * *p* < 0.05, ** *p* < 0.01 vs. the Mod group.

As shown in Figure 18, the expression levels of NLRP3, ASC, and Caspase-1 proteins were significantly elevated (*p* < 0.01) in the Mod group compared to the Con group. Treatment with allopurinol (Pos group) and high-dose ACP-60 (ACP-60-H) resulted in a significant reduction (*p* < 0.01) in the expression levels of these proteins when compared to the Mod group. Moderate-dose ACP-60 (ACP-60-M) treatment also caused a significant decrease in NLRP3 and ASC protein expression (*p* < 0.05) and an extremely significant reduction in Caspase-1 expression (*p* < 0.01). The low-dose ACP-60 (ACP-60-L) showed a significant trend of reduction or no significant change (*p* < 0.05). These findings demonstrate that ACP-60, particularly at high doses, effectively inhibits the NLRP3/ASC/Caspase-1 signaling pathway, highlighting its therapeutic potential in reducing inflammation and alleviating kidney damage in GN.

### 2.14. Effect of ACP-60 on Rats’ mRNA Expression of NLRP3, ASC, and Caspase-1

**Figure 19:**
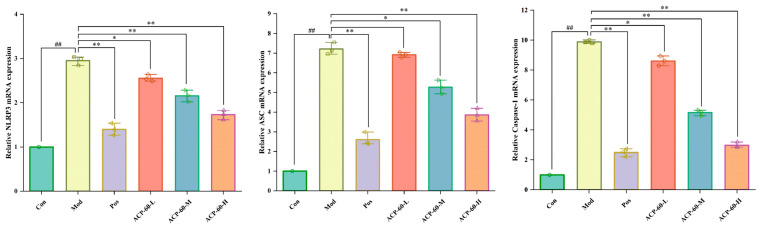
mRNA expression of NLRP3, ASC and Caspaes-1 in renal tissue. ($\overset{-}{x}$ ± s; *n* = 3) ^##^ *p* < 0.01 vs. the Con group, * *p* < 0.05, ** *p* < 0.01 vs. the Mod group.

In comparison to the Con group, the mRNA expression levels of NLRP3, ASC, and Caspase-1 in the renal tissue of the Mod group were significantly elevated (*p* < 0.01), as shown in Figure 19. Treatment with allopurinol (Pos group) and high-dose ACP-60 (ACP-60-H) resulted in a highly significant reduction in the expression of these mRNAs (*p* < 0.01) compared to the Mod group. Moderate-dose ACP-60 (ACP-60-M) significantly decreased the mRNA expression of NLRP3 and Caspase-1 (*p* < 0.01), while ASC mRNA levels were significantly reduced (*p* < 0.05). In the low-dose ACP-60 group (ACP-60-L), the mRNA expression of NLRP3 and Caspase-1 was significantly reduced (*p* < 0.05), while ASC mRNA expression showed a decreasing trend but did not reach statistical significance. These findings demonstrate that ACP-60-H has the strongest inhibitory effect on the mRNA expression of NLRP3, ASC, and Caspase-1.

## 3. Materials and Methods

### 3.1. Materials and Reagents

A total of 36 male Sprague-Dawley (SD) rats weighing 180–220 g were procured from Liaoning Changsheng Biotechnology Co., Ltd. (Benxi, China; SCXK 2020-0001). The experimental protocol was reviewed and approved by the Laboratory Animal Ethics Committee of Jiamusi University (Approval No. JDYXY-2024011).

*Atractylodes chinensis* (DC.) Koidz. samples were cultivated in the Inner Mongolia Province. Monosaccharide standards, including D-glucose (Glc) and D-galactose (Gal), were purchased from Biotopped (Beijing, China). D-xylose (Xyl), L-rhamnose (Rha), and D-fructose (Fru) were obtained from Shanghai Yuanye Bio-Technology Co., Ltd. (Shanghai, China). Antibodies specific to NLRP3, ASC, and Caspase-1 were supplied by Proteintech (Wuhan, China). Distilled water was used for all experiments, and all chemicals and reagents employed were of analytical grade.

### 3.2. Extraction and Isolation of Polysaccharides

The dried rhizome of *Atractylodes chinensis* (DC.) Koidz. was pulverized and defatted using 70% ethanol. The remaining residues were subsequently extracted with deionized water. The water-based extracts were collected, filtered, concentrated, precipitated, centrifuged, and lyophilized [30]. The crude polysaccharides were labeled as ACP after deproteinization using the Sevage method. Precipitates formed at each step during graded ethanol precipitation were collected and centrifuged. Six polysaccharide fractions, precipitated at ethanol concentrations of 30%, 40%, 50%, 60%, 70%, and 80%, were designated as ACP-30, ACP-40, ACP-50, ACP-60, ACP-70, and ACP-80, respectively. To identify the optimal polysaccharide fraction with anti-GN effects, preliminary experiments using HK-2 cells were conducted. Cell viability was assessed using the CCK-8 assay, and cell morphology was observed following ACP-30 to ACP-80 treatments. Subsequently, the protein expression levels of NLRP3, ASC, and Caspase-1 were evaluated. Among the fractions, ACP-60 demonstrated the highest activity. Based on these results, ACP-60 was selected for further experimental studies.

### 3.3. Purification and Structural Analysis of ACP-60

#### 3.3.1. Purification of ACP-60

ACP-60 was purified using a DEAE-52 cellulose column. The mobile phase consisted of 0, 0.1, and 0.2 mol/L NaCl, which were used sequentially to elute the sample at a flow rate of 1 mL/min. The phenol–sulfuric acid method was employed to determine the polysaccharide content and construct the elution curve. Following freeze-drying, three purified polysaccharide fractions (ACP-60-A, ACP-60-B, ACP-60-C) were obtained [31].

#### 3.3.2. Molecular Weight Determination

The 2 mg samples of dextran (Mw = 3 kDa–150 kDa) were accurately weighed, dissolved in water to prepare a 1 mg/mL standard solution, and filtered. ACP-60-A, ACP-60-B, and ACP-60-C were processed following the same procedure.

Conditions: Instrument: Agilent 1260Ⅱ HPLC; Detector: Shanghai Tongwei UM4800 evaporation light detector; chromatographic column: Ultrahydrogel^TM^ (Linear) water-soluble gel column (7.8 mm × 300 mm); injection volume: 10 μL.

#### 3.3.3. Monosaccharide Composition Analysis

ACP-60-A, ACP-60-B, and ACP-60-C (5 mg each) were hydrolyzed in 2 mL of 2M trifluoroacetic acid (TFA) solution for 2 h at 120 °C. The monosaccharide standards were also processed. After processing, the samples were analyzed by high-performance liquid chromatography (HPLC) with an evaporative light scattering detector (ELSD).

Conditions: Instrument: Agilent 1260Ⅱ HPLC; Column: Waters XBridge^TM^ Amide (250 mm × 4.6 mm, 5 µm); Detector: evaporative light scattering detector; column temperature: 30 °C; flow rate: 1 mL/min; sample intake: 10 μL.

#### 3.3.4. Infrared Spectroscopy (IR) Analysis

ACP-60-A, ACP-60-B, and ACP-60-C (2 mg each) were mixed and crushed with dry KBr powder. The samples were then scanned using a Fourier-transform infrared spectrometer (FTIR-650, Bruker, Rheinstetten, Germany) at frequencies between 500 and 4000 cm^−1^ [32].

#### 3.3.5. Methylation Analysis

Slight modifications were made to previously described methods. The lyophilized ACP-60-A sample was analyzed for methylation using a GC-MS system (Agilent 7890A-5975C, Santa Clara, CA, USA) with a DB-5 silica column (60 m × 0.25 mm × 0.25 μm).

Conditions: Inlet temperature: 250 °C; the shunt ratio: 1:20; column flow rate: 1.2 mL/min; scan range: *m*/*z* 43–500; scan rate: 2.5 scans/s [33].

#### 3.3.6. NMR Analysis

ACP-60-A was dissolved in D_2_O and placed in an NMR tube for analysis using an NMR instrument (Bruker AM-400, Bruker, Fällanden, Switzerland). Both one-dimensional (1D) and two-dimensional (2D) NMR spectra were recorded and analyzed.

### 3.4. Analysis of the Anti-Gouty Nephropathy Activity of ACP-60-A, ACP-60-B and ACP-60-C

#### 3.4.1. Determination of HK-2 Cell Viability

HK-2 cells were divided into five groups: Con, Mod, ACP-60-A, ACP-60-B, and ACP-60-C. Except for the Con group, all groups were treated with 800 μmol/L UA in the culture medium. The ACP-60-A, ACP-60-B, and ACP-60-C groups were additionally treated with 80 μg/mL of their respective compounds. HK-2 cells (10^5^ per well) were cultured in 96-well plates at 37 °C in a 5% CO_2_ incubator. The CCK-8 assay was used to evaluate cell viability (SpectraMax ABS Plus, Molecular Devices, San Jose, CA, USA) [34].

#### 3.4.2. Morphological Observation of HK-2 Cells

HK-2 cells were cultured at 37 °C in a 5% CO_2_ incubator. Following treatment, UA-containing medium and ACP-60-A, ACP-60-B, andACP-60-C were added to the respective groups. After 36 h of incubation, morphological changes in the cells were observed under an inverted microscope and documented through photomicrography.

#### 3.4.3. Western Blotting Analysis

The protein expression levels of NLRP3, ASC, and Caspase-1 were analyzed in HK-2 cells from each group. Protein concentrations were determined using a bicinchoninic acid (BCA) protein assay kit. Proteins were incubated overnight at 4 °C with specific antibodies against NLRP3, ASC, Caspase-1, and β-actin [35]. The resulting blots were visualized using Lab imaging software (Tanon-4200, 2.6.0.0, Tanon, Shanghai, China).

### 3.5. Analysis of the Anti-Gouty Nephropathy Activity of ACP-60

#### 3.5.1. Gouty Nephropathy Model Establishment

The experimental GN model was established by modifying a previously reported method [5]. A week before the experiment, the rats were allowed to acclimate in a controlled setting with unrestricted access to food and water. After acclimatization, the SD rats were randomly divided into six groups (*n* = 6/group): control group (Con, saline), model group (Mod, adenine), positive control group (Pos, allopurinol, 40 mg/kg), and low-, medium-, and high-dose ACP-60 groups (ACP-60-L, 60 mg/kg; ACP-60-M, 120 mg/kg; ACP-60-H, 240 mg/kg).

Adenine suspension (100 mg/kg/day) was administered intragastrically to all groups except the Con group, and a high-yeast diet (10 mg/kg/day) was provided for 35 days to induce the GN model. From day 8 onward, saline was administered to the Con group, while other groups received their respective treatments in the afternoon for 28 days. Body weights were recorded every two days throughout the study.

#### 3.5.2. Determination of 24-h Urinary Protein

On day 35, 24-h urine samples were collected from each group using metabolic cages. Samples were centrifuged, and the supernatant was analyzed for urinary protein content using a Coomassie Brilliant Blue (CBB) reagent kit.

#### 3.5.3. Determination of Serum Biochemical Parameters

Following anesthesia with sodium pentobarbital, blood was collected from the abdominal aorta of each rat. The blood was centrifuged at 3500 rpm for 10 min at 4 °C, and serum supernatant was analyzed for levels of UA, Cr, and BUN using a reagent kit.

#### 3.5.4. Determination of IL-18 and IL-1β

Renal tissue samples were processed, and their supernatants were collected. Levels of IL-1β and IL-18 were measured using ELISA kits based on provided instructions [36].

#### 3.5.5. Renal Histopathological Analysis

The left kidneys of the rats were rinsed in saline, fixed, embedded, sectioned, and stained with hematoxylin and eosin (HE). Histomorphological changes were observed and analyzed under a microscope.

#### 3.5.6. Western Blotting Analysis

Total protein was extracted from kidney tissue following a previously described method [37]. Tissue samples were homogenized, washed, lysed with radioimmunoprecipitation assay (RIPA) buffer, and centrifuged. Protein concentrations were determined using a bicinchoninic acid (BCA) protein assay kit. Proteins were incubated overnight at 4 °C with specific antibodies against NLRP3, ASC, Caspase-1, and β-actin. The resulting blots were visualized using Lab imaging software.

#### 3.5.7. qRT-PCR Analysis

TRIzol reagent and a cDNA reverse transcription kit were used to extract total RNA and cDNA from kidney tissue, respectively. For the qRT-PCR reaction, 0.2 mL PCR tubes were loaded with a reaction mix comprising 7.5 μL of 2 × Universal Blue SYBR Green qPCR Master Mix, 1.5 μL of 2.5 μM gene primer (upstream + downstream), 2.0 μL of cDNA, and 4.0 μL of nuclease-free water. The PCR tube was placed in the PCR apparatus for amplification. Based on the cycle threshold (CT) values, the relative expression levels of target genes in the samples were measured [38]. A listing of the primer set sequences used for amplification may be found in Table 5.

### 3.6. Data Analysis

The means ± standard errors of the means ($\overset{-}{x}$ ± s) were used to display the data. SPSS 23.0 software was used to process data using the *t*-test. A difference was deemed statistically significant if it was *p* < 0.05 or *p* < 0.01.

## 4. Conclusions and Discussion

In this study, the sub-fractions were extracted using graded ethanol, and the optimal fraction was identified through screening with HK-2 cells. Based on these results, ACP-60 was selected for further experimental studies. Using a DEAE-52 cellulose column, ACP-60 was separated and purified into three fractions: ACP-60-A, ACP-60-B, and ACP-60-C. The FT-IR results showed that ACP-60-A, ACP-60-B, and ACP-60-C had characteristic absorption bands of polysaccharides, but ACP-60-A contained a small amount of proteinaceous and polyphenolic impurities. The molecular weights of these fractions were determined to be 9.18 kDa, 58.21 kDa, and 109.01 kDa, respectively. Structural characterization of the fractions was performed by analyzing their monosaccharide compositions and IR spectra. Monosaccharide analysis revealed that ACP-60-A primarily consisted of Glc and Fru. ACP-60-B contained Rha, Gal, Fru, and Man, while ACP-60-C was mainly composed of Man, Rha, Gal, and Xyl.

The anti-GN effects and mechanisms of ACP-60-A, ACP-60-B, and ACP-60-C were further investigated using HK-2 cells. Experimental results demonstrated that all three fractions exhibited anti-GN activity, consistent with prior pharmacological studies. Among these, ACP-60-A displayed superior anti-GN activity. Consequently, ACP-60-A underwent methylation and NMR analysis to elucidate its glycosidic bond connections and explore its structure-activity relationship. Three glycosidic bonds were identified in ACP-60-A, including Glc*p*-(1→, β-Fru*f*-(2→ and →1)-Fru*f*-(2→. Since →1)-Fru*f*-(2→ constitutes a significant proportion of the total sugar residues, it is hypothesized that the repeating unit backbone of ACP-60-A consists of 1,2-linked-Fru*f*. Further NMR analysis confirmed that ACP-60-A is primarily composed of α-Glc*p*-(1→, →2)-β-Fru*f* and →1)-β-Fru*f*-(2→. Thus, it is proposed that ACP-60-A features a →1)-β-Fru*f*-(2→ main chain with α-Glc*p*-(1→ and β-Fru*f*-(2→ linked at its termini.

The structure-activity relationship analysis suggests that ACP-60-A’s superior activity may stem from differences in molecular weight, monosaccharide composition, and the specific arrangement of glycosidic bonds. Polysaccharide activity is influenced by factors such as molecular weight, monosaccharide composition, glycosidic bonds, and structural configuration [39,40]. Lower molecular weight polysaccharides tend to exhibit enhanced biological activity due to their ability to cross cell membranes more readily and induce biological responses. Additionally, variations in monosaccharide composition significantly impact biological activity, and the different monosaccharide compositions of ACP-60-A, ACP-60-B, and ACP-60-C had a greater impact on biological activity. Studies have reported that inulin-type fructans isolated from Codonopsis polysaccharides exhibit potent anti-inflammatory effects. Similarly, polysaccharides with higher Fru content have shown improved immunomodulatory and antioxidant properties [41,42]. A study investigating the inulin-type fructans with different molecular weights showed that only the short-chain fructans possessed anti-inflammatory properties, which might be related to the structure of fructans and the chain length [43]. Therefore, ACP-60-A is likely to exhibit superior bioactivity compared to ACP-60-B and ACP-60-C, attributed to its lower molecular weight, elevated Fru content, and distinct glycosidic bond arrangement within its structure.

This study provides a comprehensive understanding of the pathogenic mechanisms of GN, particularly the role of the NLRP3 inflammasome in the progression of kidney disease. The NLRP3 inflammasome, an inflammatory complex composed of NLRP3, ASC, and Caspase-1, plays a pivotal role in promoting inflammation by activating the NLRP3/ASC/Caspase-1 signaling pathway. Activation of NLRP3 leads to the activation of Caspase-1, which subsequently induces the release of pro-inflammatory cytokines IL-1β and IL-18. Previous studies have demonstrated that suppressing the inflammatory response can delay GN progression [44,45,46,47]. The rhizome of Atractylodes chinensis (DC.) Koidz. has been used as a traditional herbal medicine against digestive And inflammatory disorders. Atractylodes chinensis (DC.) Koidz. polysaccharides (AKPs) have shown hypoglycemic activity, and their effects on ameliorating the ecological dysregulation of gut microbes in patients with type 2 diabetes mellitus (T2DM) were investigated using an in vitro simulated digestive fermentation model [20]. Moreover, AKP extraction using the ultrasonic method could improve the yield of AKUs, increase the sugar content, and show synergistic anti-gastric cancer effects in combination with oxaliplatin [19]. In this study, polysaccharides were extracted from the traditional Chinese medicinal plant *Atractylodes chinensis* (DC.) Koidz. and the anti-GN effects of varying doses of ACP-60 were evaluated in SD rats. The results indicate that ACP-60 improved kidney function, which may be associated with modulation of the NLRP3 inflammasome signaling pathway. Specifically, ACP-60 appeared to inhibit the assembly of the NLRP3 inflammasome and reduce IL-1β and IL-18 levels.

Serum levels of UA, BUN, and Cr were employed as key indicators of renal function. Elevated UA levels contribute to renal tubular deposition, leading to kidney damage, reduced glomerular filtration, and diminished excretion of BUN and Cr, thereby causing their accumulation in the blood. These processes collectively promote the onset of GN [48]. Treatment with ACP-60 significantly decreased serum levels of UA, BUN, and Cr in GN rats, with the high-dose group (ACP-60-H) showing the most pronounced reductions compared to the low- and medium-dose groups (ACP-60-L and ACP-60-M).

Histopathological analysis of renal tissue revealed that ACP-60-H markedly improved glomerular morphology and renal structural integrity. Additionally, ACP-60-H reduced renal fibrosis and mitigated inflammatory infiltration in the renal interstitial regions. These findings suggest that ACP-60-H provides nephroprotection comparable to the commercially available drug allopurinol. Moreover, ACP-60-H exhibited superior nephroprotective activity and a greater ability to suppress inflammatory responses. In conclusion, ACP-60, particularly at high doses, shows promise as a therapeutic agent for GN by improving renal function, reducing inflammation, and protecting against kidney damage.

IL-1β, a key inflammatory mediator in GN, is predominantly produced via the NLRP3 inflammasome signaling pathway. Activation of NLRP3 leads to the activation of Caspase-1, which converts Pro-IL-1β into its mature form, IL-1β, subsequently released into the extracellular environment. In this study, ACP-60 demonstrated the ability to reduce the inflammatory cytokines IL-18 and IL-1β. Experimental findings revealed that ACP-60 not only downregulated the expression of NLRP3, ASC and Caspase-1 proteins but also inhibited their mRNA expression levels.

**Figure 20:**
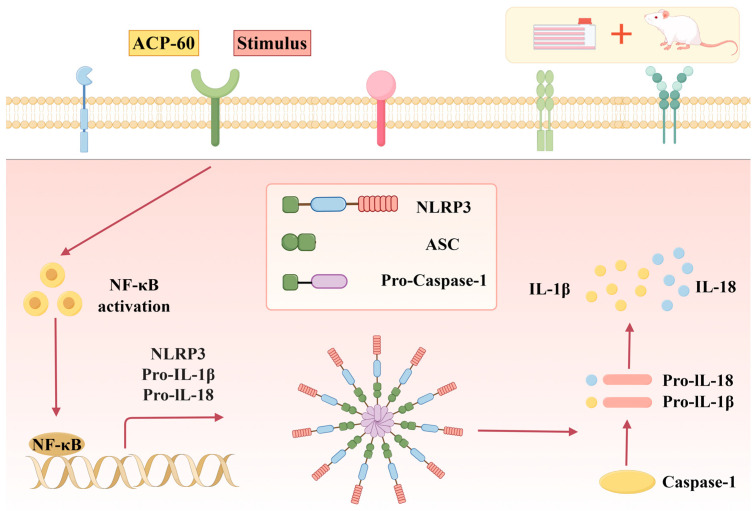
Diagram of the mechanism of ACP-60 (drawn using figdraw).

These results suggest that ACP-60 alleviates GN progression by suppressing the NLRP3 inflammasome, thereby reducing the secretion of downstream inflammatory cytokines. It is hypothesized that this mechanism of action, mediated through the inhibition of NLRP3, ASC, and Caspase-1 signaling, leads to a decrease in IL-18 and IL-1β production and release, ultimately mitigating renal injury in GN rats. The proposed mechanism is illustrated in Figure 20.

In conclusion, this study provides the first evidence that ACP offers kidney protection in GN rats, potentially through the inhibition of the NLRP3/ASC/Caspase-1 signaling pathway. By elucidating the structural characteristics and preliminary pharmacological mechanisms of ACP-60, this research establishes a foundation for its scientific development and utilization in GN treatment.

With the increased complexity of GN and the diversification of drug targets, the difficulty and cost of developing anti-GN drugs in the future are rising. Similarly, the development of ACP-60 drug against GN also faces multiple difficulties; it requires a large capital investment and time cost and needs to undergo several rigorous clinical trials. Moreover, multiple departments are needed to ensure its safety and efficacy feasibility before it can be used. In addition, ACP-60 should be used with other drugs to avoid new side effects and ensure its safety. Further purification or modification of ACP-60 may enhance its anti-GN efficacy, paving the way for the development of safer and more effective herbal medicines for GN management in the future.

## Figures and Tables

**Table 1:** Extraction rate and sugar contents of ACP-30-80.

	ACP-30	ACP-40	ACP-50	ACP-60	ACP-70	ACP-80
Extraction rate (wt%)	7.63 ± 1.21	12.16 ± 1.34	10.05 ± 1.18	20.89 ± 2.13	39.06 ± 1.95	10.21 ± 1.52
Sugar content (%)	57.45 ± 1.82	75.16 ± 2.15	73.07 ± 1.79	77.21 ± 2.41	74.81 ± 2.37	72.44 ± 1.91

**Table 2:** Monosaccharide composition analysis of ACP-60-A, ACP-60-B, and ACP-60-C.

Sample	Rha	Fuc	Xyl	Ara	Fru	Man	Glc	Gal
ACP-60-A	-	-	-	-	1.00	-	0.15	-
ACP-60-B	0.05	-	-	-	0.24	0.14	-	0.20
ACP-60-C	0.11	-	0.53	-	-	0.29	-	1.04

**Table 3:** Methylation analysis results of ACP-60-A.

**Deduced Residues**	**Derivatives**	**mol%**
Fru*f*-(2→	2,5-Di-O-acety-(2-deuterio)-1,3,4,6-tetra-O-methyl hexitol (mannitol, glucitol)	23.38
Glc*p*-(1→	1,5-Di-O-acetyl-(1-deuterio)-2,3,4,6-tetra-O-methyl glucitol	15.90
→1)-Fru*f*-(2→	1,2,5-Tri-O-acetyl-(2-deuterio)-3,4,6-tri-O-methyl hexitol (mannitol, glucitol)	60.72

**Table 4:** ^1^H/^13^C chemical shifts of ACP-60-A from HSQC data.

Sugar Residues	Chemical Shifts, *δ* (ppm)					
	H1/C1	H2/C2	H3/C3	H4/C4	H5/C5	H6/C6
→1)-β-Fru*f*-(2→A	3.85, 3.62/60.87	-/103.67	4.18/76.97	4.02/74.26	3.77/81.05	3.75, 3.68/62.11
α-Glc*p*-(1→B	5.36/92.46	3.45/71.16	3.68/72.58	3.39/69.21	3.76/72.41	3.66/60.45
β-Fru*f*-(2→C	3.85, 3.62/60.87	-/103.21	4.18/76.97	4.02/74.26	3.77/81.05	3.75, 3.68/62.11

**Table 5:** Real-time quantitative PCR primer sequences.

**Gene**	**Primary Sequence**
NLRP3	5′-GATTTCTCCACAACTCACCCAA-3′ 5′-AGTCTGGAAGAACAGGCAACAT-3′
ASC	5′-ACTATCTGGAGGGGTATGGCTT-3′ 5′-CAATGAGTGCTTGCCTGTGTT-3′
Caspase-1	5′-TGCCTGGTCTTGTGACTTGGAG-3′ 5′-TGTCCTGGGAAGAGGTAGAAACG-3′
GADPH	5′-CTGGAGAAACCTGCCAAGTATG-3′ 5′-GGTGGAAGAATGGGAGTTGCT-3′

## Data Availability

Data are contained within the article.

## Abbreviations

ACP, *Atractylodes chinensis* (DC.) Koidz Polysaccharides; GN, Gouty nephropathy; UA, uric acid; NLRP3, (NOD)-like receptor protein 3; ASC, apoptosis-associated speck-like protein containing a CARD; SD, Sprague-Dawley; Cr, creatinine; IL-1β, interleukin 1β; IL-18, interleukin 18; BCA, bicinchoninic acid; SEM, scanning electron microscope.
